# Supplementary material for: Programmed spatial organization of biomacromolecules into discrete, coacervate-based protocells
Source: Nat Commun. 2020 Dec 8;11:6282. doi: 10.1038/s41467-020-20124-0 (PMC7722712; doi:10.1038/s41467-020-20124-0)
Supplement: Supplementary file 1 — Supplementary Information [file 41467_2020_20124_MOESM1_ESM.pdf]

Supplementary Information

Programmed spatial organization of biomacromolecules into discrete, coacervate-based protocells

Wiggert J. Altenburg[1,2], N. Amy Yewdall[1,2], Daan F.M. Vervoort[1,2], Marleen H.M.E. van Stevendaal[2,3], Alexander F. Mason[2, 3]* & Jan C. M. van Hest[1,2,3]*

[1] Department of Biomedical Engineering, Eindhoven University of Technology, PO Box 513, 5600 MB, Eindhoven, the Netherlands

[2] Institute for Complex Molecular Systems, Eindhoven University of Technology, PO Box 513, 5600 MB, Eindhoven, the Netherlands

[3] Department of Chemical Engineering and Chemistry, Eindhoven University of Technology, PO Box 513, 5600 MB, Eindhoven, the Netherlands

Correspondence should be addressed to a.f.Mason@tue.nl or j.c.m.v.Hest@tue.nl

Supplementary Notes:

Chemicals and NMR Spectroscopy. All chemicals were used as received unless otherwise stated. For the synthesis of terpolymer: monomethoxy poly(ethylene glycol) 1 and 2 kDa were purchased from Rapp Polymere, trimethylene carbonate was purchased from TCI Europe. For the preparation of modified amylose derivatives: amylose (12-16 kDa) was supplied by Carbosynth and 3-chloro-2-hydroxypropyltrimethyl ammonium chloride (65 wt% in water) was supplied by TCI Europe. NMR spectra were recorded in the specified deuterated solvents (Cambridge Isotope Laboratories) using a Bruker Avance III 400 MHz NMR spectrometer. Chemical shifts are measured in parts per million (ppm), internally referenced relative to tetramethylsilane.

Synthesis of modified amyloses. Both quaternized (Q-Am) and carboxymethylated (CM-Am) amylose were prepared in accordance with previously published procedures.[1]

Q-Am was prepared by dissolving 1.5 g amylose and 2.78 g NaOH in 14.25 mL Milli-Q at 35 °C. After complete dissolution of the amylose, 11.64 mL 3-chloro-2-hydroxypropyltrimethylammonium chloride solution (60 wt% in water) was added dropwise into the stirring reaction mixture, which was subsequently left to react overnight. After this time, the mixture was neutralized with acetic acid and precipitated into 200 mL cold ethanol. The resulting precipitate was re-dissolved in Milli-Q water and dialyzed extensively against water using regenerated cellulose dialysis tubing (Spectrum Labs, USA) with a 3.5 kDa MWCO before lyophilization. Around 5 g of Q-Am, with a degree of substitution of 0.88, was obtained from this reaction (ca. 80 % yield). ^1H NMR (D_2O) characterization data are presented in Supplementary Figure 1a.

Cm-Am was prepared by dissolving 1.5g amylose and 3.6 g NaOH in 15 mL Milli-Q at 70 °C. After complete dissolution of the amylose, 2.7 g chloroacetic acid was added and the reaction mixture was left to stir for 2 h. After the reaction, the mixture was neutralized with acetic acid and precipitated into 200 mL cold ethanol. The resulting precipitate was re-dissolved in Milli-Q water and dialyzed extensively against water using regenerated cellulose dialysis tubing (Spectrum Labs, USA) with a 3.5 kDa MWCO before lyophilization. Around 5 g Cm-Am, with a degree of substitution of 0.4, was obtained from this reaction (ca. 80 % yield). ^1H NMR (D_2O) characterization data are presented in Supplementary Figure 1b.

Synthesis of copolymer. Copolymer was synthesized in accordance with previously published procedures.[1] Step 1 - Preparation of poly(ethylene glycol)-poly(ε-caprolactone-gradient-trimethylene carbonate) (PEG-PClgTMC). According to a modified literature procedure the organocatalyzed ring-opening polymerization of ε-caprolactone and trimethylene carbonate was performed, aiming for a composition of PEG_{44}-PCL_{50}-g-TMC_{50}.[2] Monomethoxy-PEG-OH macroinitiator (2 kDa, 0.2 mmol, 400 mg) was weighed into a round-bottomed flask along with ε-caprolactone (ε-CL, 10 mmol, 1108 µL) and trimethylene carbonate (TMC, 10 mmol, 1121 mg) and dried via azeotropic evaporation of added toluene (x3). The dried reagents were then re-dissolved in dry toluene (20 mL) and methanesulfonic acid (0.6 mmol, 39 µL) was added, under argon. The reaction mixture was stirred at 30 °C for 6 h, after which time it was precipitated into ice cold methanol. This yielded 2.1 g of a waxy solid (85 % yield) and the composition of the resulting copolymer was confirmed by ^1H NMR ($CDCl_3$), comparing the protons of PEG (3.65-3.7 ppm), terminal methyl unit (singlet at 3.40 ppm) to PCL CH_2 (multiplet at 2.40-2.25 ppm) and PTMC CH_2 (multiplet at 2.2-1.8 ppm). GPC analysis (using a PL gel 5 µm mixed D column, with THF and PS standards) yielded a Đ of 1.1.

Step 2 - Chain-end modification with Boc-L-phenylalanine and deprotection. For the incorporation of a terminal amine onto the diblock copolymer, we performed a carbodiimide-mediated esterification between PEG-PCLgTMC and Boc-L-phenylalanine (Boc-L-Phe). 1.3 g (ca. 0.1 mmol) of PEG-PCLgTMC

copolymer was dissolved in acetonitrile and to it was added *N*,*N′*-dicyclohexylcarbodiimide (0.2 mmol, 41.9 mg), Boc-L-Phe (0.5 mmol, 23.4 mg), and 4-dimethylaminopyridine (0.02 mmol, 2.5 mg) on ice. The reaction mixture was stirred for 24 h at RT and afterwards placed directly in the freezer overnight to facilitate precipitation of dicyclohexylurea by-product. After cold filtration of the reaction mixture it was concentrated and then precipitated into cold methanol. ^1H NMR ($CDCl_3$) was used to check the product to confirm disappearance of the terminal TMC signal at 1.92 ppm due to addition of phenylalanine at the terminus, and emergence of aromatic protons at around 7.2-7.3 ppm and Boc protons at 1.45 ppm before deprotection. The resulting copolymer was then dissolved in 5 mL DCM, to which 5 mL of trifluoro acetic acid (TFA) was added (on ice) and the mixture was allowed to warm to RT and stirred for 2 h. After 2 h the solvent was evaporated and the copolymer was washed with $NaHCO_3$, 1 M NaCl and brine before drying on $MgSO_4$, filtration and evaporation of the majority of the solvent so that the copolymer could be precipitated into ice cold methanol. ^1H NMR ($CDCl_3$) clearly showed that the signal arising from the Boc group had disappeared. GPC analysis before and after deprotection yielded a Đ of 1.1, indicating that TFA treatment did not induce copolymer hydrolysis.

Step 3 - Polymerization and deprotection of *N*-carboxyanhydride γ-benzyl-L-glutamate (NCA-BLG). Following a published method for the controlled polymerization of NCA-BLG by lowering the temperature and maintaining a constant flow of N_2 in order to remove CO_2 by-product,[3] we aimed to add between 8 and 10 PBLG units onto the amine-terminus of the copolymer chains. 1 g of Phe-terminated copolymer was weighed into a Schlenk flask and dissolved with ca. 3 mL of dry DMF and cooled in an ice bath. To the cooled mixture 160 mg of NCA-BLG was added under Ar and the reaction was left under a constant flow of N_2 for 24 h. The product was precipitated into cold methanol and analysed by ^1H NMR ($CDCl_3$) to confirm the overall composition and, in particular, the presence of benzylic and aromatic protons at 5.0-5.2 and 7.1-7.4 ppm, respectively. Benzyl-protected terpolymer was dissolved in 10 mL THF and 10 mL methanol was added before applying hydrogenation using the H-Cube at 60 °C with 30 bar of H_2 pressure and a flow rate of 1 mL min^{-1} to facilitate removal of the benzyl protecting groups. The product was concentrated, then precipitated into cold ether, and dissolved in dioxane before lyophilisation to yield a waxy solid, 0.9 g (85 % yield). ^1H NMR ($CDCl_3$) was used to confirm successful deprotection of the PBLG units, and GPC data indicated that the polydispersity did not increase beyond 1.1 during this process.

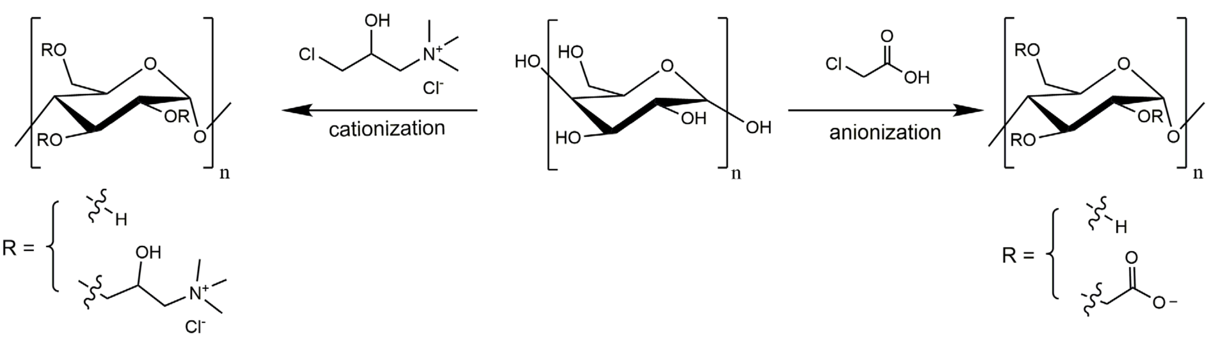

Supplementary Figure 1: Modification of amylose with either 3-chloro-2-hydroxypropyltrimethylammonium chloride solution or chloroacetic acid to form Q-Am and Cm-Am, respectively.

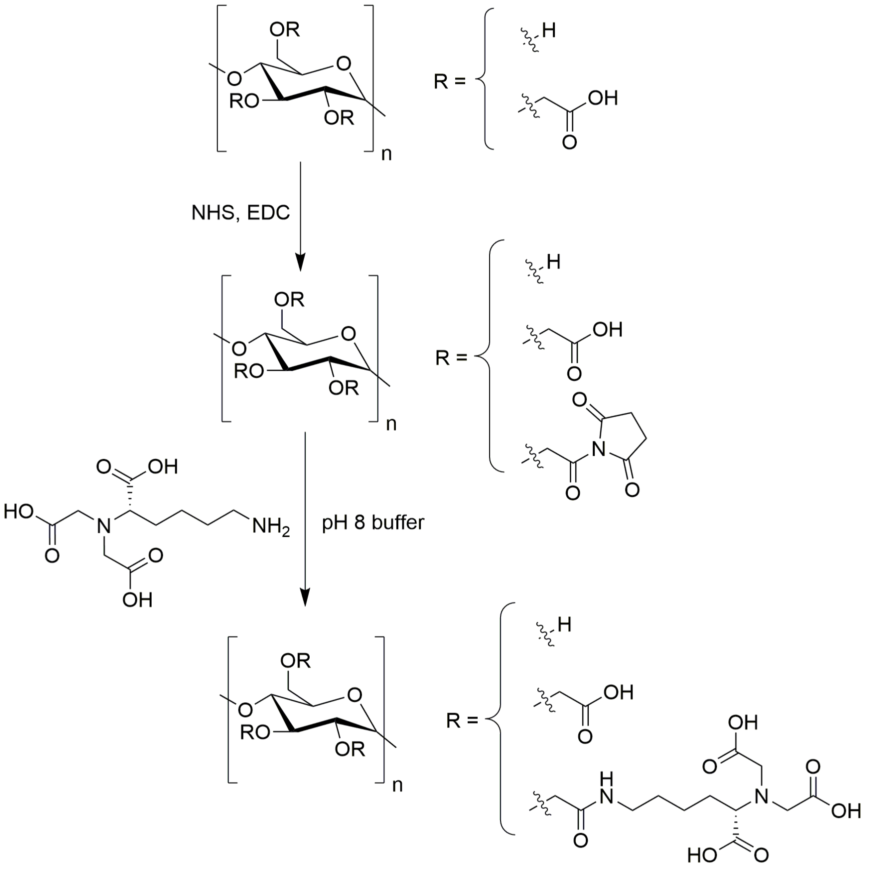

Supplementary Figure 2: Synthesis of NTA-Am from Cm-Am by EDC/NHS followed by amide bond formation with an amine-functionalized NTA.

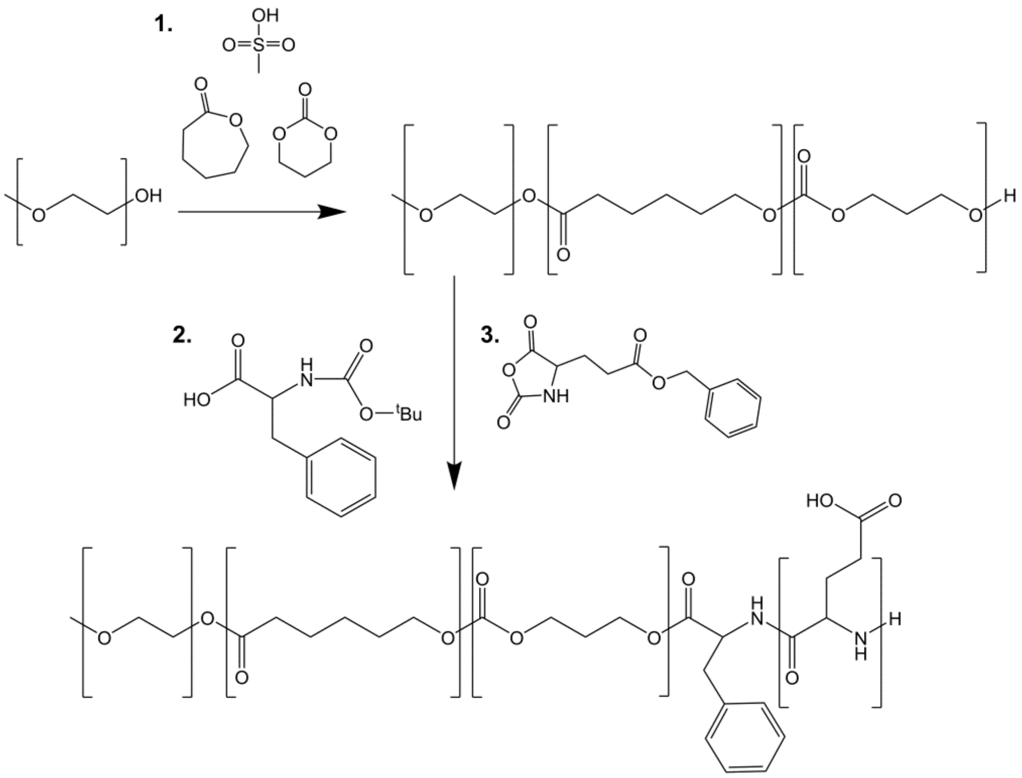

Supplementary Figure 3: Synthesis of PEG-b-PCLgPTMC-b-PGA terpolymer. Poly(ethylene glycol) monomethyl ether was used to initiate the ring opening polymerisation of ε-caprolactone and trimethylene carbonate (step 1). The terminal alcohol of this polymer was subsequently modified via a Steglich esterification with Boc-L-Phe-OH to yield a primary amine after TFA deprotection (step 2). The final poly(L-glutamic acid) block was introduced by the ring opening polymerisation of N-carboxyanhydride γ-benzyl-L-glutamate, followed by hydrogenation (step 3)

Supplementary table 1: Sequences and parameters of the proteins used in this study. **Histidine-tag** shown in green and **TEV protease site** is represented in blue. Parameters were calculated using the online ProtParam tool (ExPASy).

**Name**	**Sequence**	**Mass:**	**Theoretical pI:**	**Additional information**
**sfGFP**	MVKMGASKGEELFTGVVPI LVELDGDVNGHKFSVRGEG EGDATNGKLTLKFICTTGKL PVPWPTLVTTLTYGVQCFS RYPDHMKQHDFFKSAMPE GYVQERTISFKDDGTYKTRA EVKFEGDTLVNRIELKGIDFK EDGNILGHKLEYNFNSHNV YITADKQKNGIKANFKIRHN VEDGSVQLADHYQQNTPI GDGPVLLPDNHYLSTQSAL SKDPNEKRDHMVLLEFVTA AGITHGMDELYKTLPETG**E NLYFQS**GGS**HHHHHH***	29.71 kDA	6.04	-
**-30GFP**	MVKMGASKGEELFDGVVP ILVELDGDVNGHEFSVRGE GEGDATEGELTLKFICTTGE LPVPWPTLVTTLTYGVQCF SDYPDHMDQHDFFKSAMP EGYVQERTISFKDDGTYKTR AEVKFEGDTLVNRIELKGID FKEDGNILGHKLEYNFNSH DVYITADKQENGIKAEFEIR HNVEDGSVQLADHYQQNT PIGDGPVLLPDDHYLSTESA LSKDPNEDRDHMVLLEFVT AAGIDHGMDELYKTLPETG **ENLYFQS**GGS**HHHHHH***	29.71 kDa	4.63	-
**+36GFP**	MVKMGASKGERLFRGKVPI LVELKGDVNGHKFSVRGKG KGDATRGKLTLKFICTTGKL PVPWPTLVTTLTYGVQCFS RYPKHMKRHDFFKSAMPK GYVQERTISFKKDGKYKTRA EVKFEGRTLVNRIKLKGRDF KEKGNILGHKLRYNFNSHK VYITADKRKNGIKAKFKIRH NVKDGSVQLADHYQQNTP IGRGPVLLPRNHYLSTRSKLS KDPKEKRDHMVLLEFVTAA GIKHGRDERYKTLPETG**ENL YFQS**GGS**HHHHHH***	30.41 kDa	10.32	-

Tryptophanase **(TnaA)**	MVKMHHHHHHGGSENLY<br>FQSGGENFKHLPEPFRIRVI<br>EPVKRTTRAYREEAIIKSGM<br>NPFLLDSEDVFIDLLTDSGT<br>GAVTQSMQAAMMRGDE<br>AYSGSRSYYALAESVKNIFG<br>YQYTIPTHQGRGAEQIYIPV<br>LIKKREQEKGLDRSKMVAFS<br>NYFFDTTQGHSQINGCTVR<br>NVYIKEAFDTGVRYDFKGN<br>FDLEGLERGIEEVGPNNVPY<br>IVATITSNSAGGQPVSLANL<br>KAMYSIAKKYDIPVVMDSA<br>RFAENAYFIKQREAEYKDW<br>TIEQITRETYKYADMLAMS<br>AKKDAMVPMGGLLCMKD<br>DSFFDVYTECRTLCVVQEGF<br>PTYGGLEGGAMERLAVGLY<br>DGMNLDWLAYRIAQVQYL<br>VDGLEEIGVVCQQAGGHA<br>AFVDAGKLLPHIPADQFPA<br>QALACELYKVAGIRAVEIGS<br>FLLGRDPKTGKQLPCPAELL<br>RLTIPRATYTQTHMDFIIEAF<br>KHVKENAANIKGLTFTYEPK<br>VLRHFTAKLKEV*	55.15 kDa	6.19	From *Escherichia coli*<br>PDB: 2C44
Flavin-containing monooxygenase **(FMO)**	MVKMHHHHHHGGSENLY<br>FQSGGATRIAILGAGPSGM<br>AQLRAFQSAQEKGAEIPELV<br>CFEKQADWGGQWNYTW<br>RTGLDENGEPVHSSMYRYL<br>WSNGPKECLEFADYTFDEH<br>FGKPIASYPPREVLWDYIKG<br>RVEKAGVRKYIRFNTAVRH<br>VEFNEDSQTFTVTVQDHTT<br>DTIYSEEFDYVVCCTGHFST<br>PYVPEFEGFEKFGGRILHAH<br>DFRDALEFKDKTVLLVGSSY<br>SAEDIGSQCYKYGAKKLISCY<br>RTAPMGYKWPENWDERP<br>NLVRVDTENAYFADGSSEK<br>VDAIILCTGYIHHFPFLNDDL<br>RLVTNNRLWPLNLYKGVV<br>WEDNPKFFYIGMQDQWY<br>SFNMFDAQAWYARDVIM<br>GRLPLPSKEEMKADSMAW<br>REKELTLVTAEEMYTYQGD<br>YIQNLIDMTDYPSFDIPATN<br>KTFLEWKHHKKENIMTFRD<br>HSYRSLMTGTMAPKHHTP<br>WIDALDDSLEAYLSDKSEIP<br>VAKEAGS*	55.50 kDa	5.40	From *Methylophaga aminisulfidivorans*<br>PDB: 2XVE

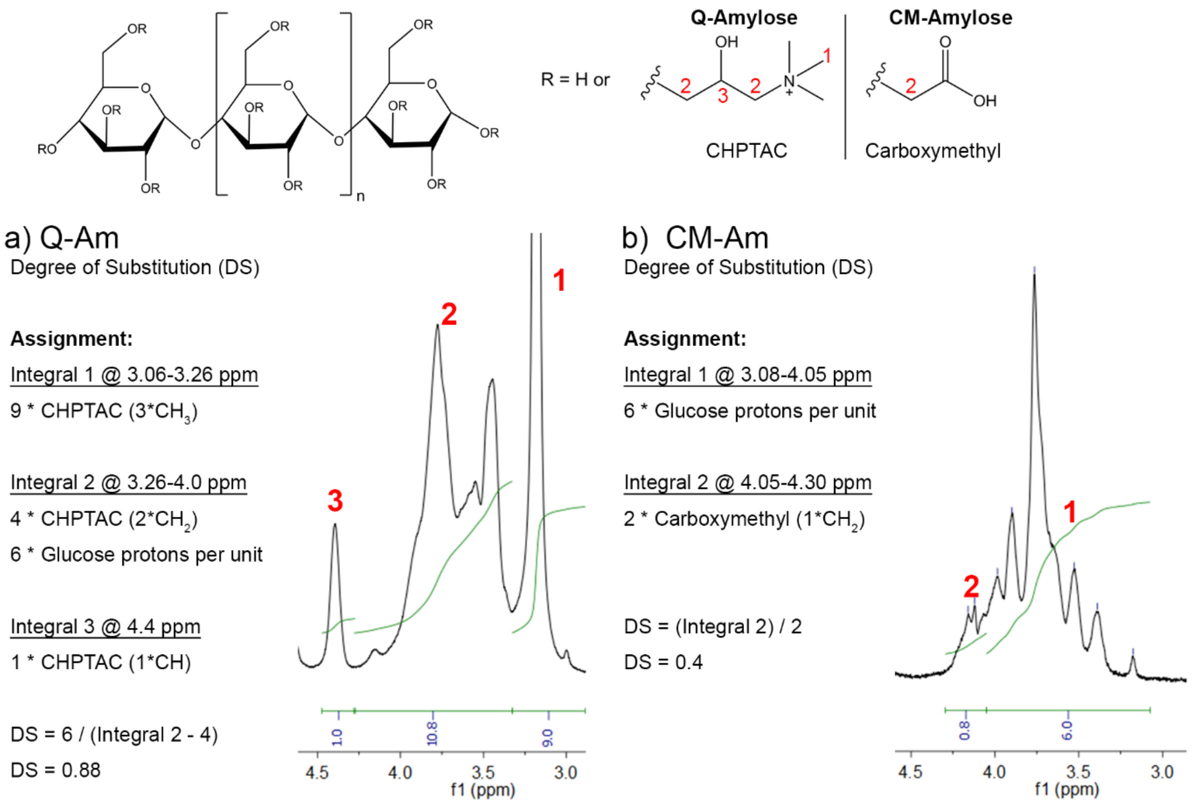

Supplementary Figure 4. ^{1}H NMR processing of highly charged amylose derivatives, including calculation of the degree of substitution (DS = number of modifying groups per glucose unit), of both quaternized (Q-Am) and carboxymethylated (Cm-Am) products.

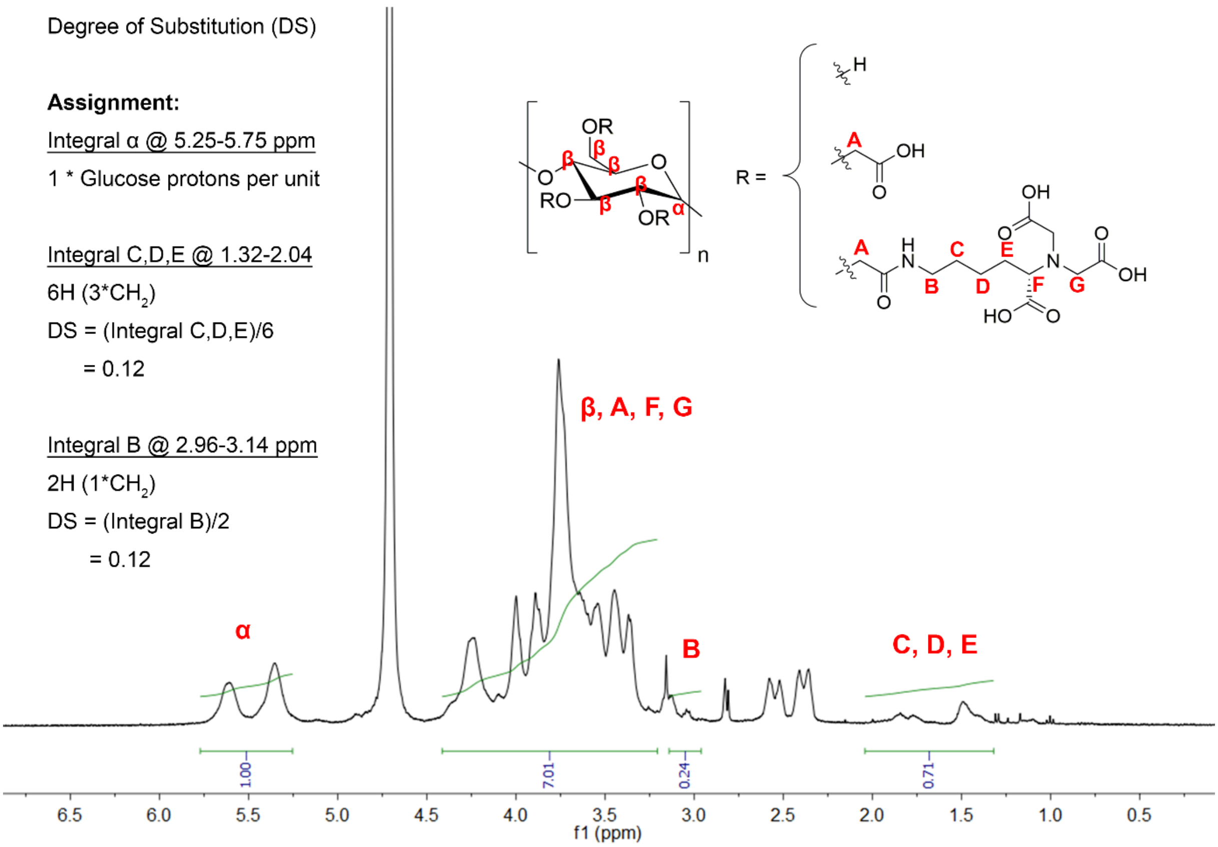

Supplementary Figure 5. ^{1}H NMR spectroscopy of NTA-Am, after extensive dialysis against ultrapure water and lyophilization, with calculation of the degree of substitution.

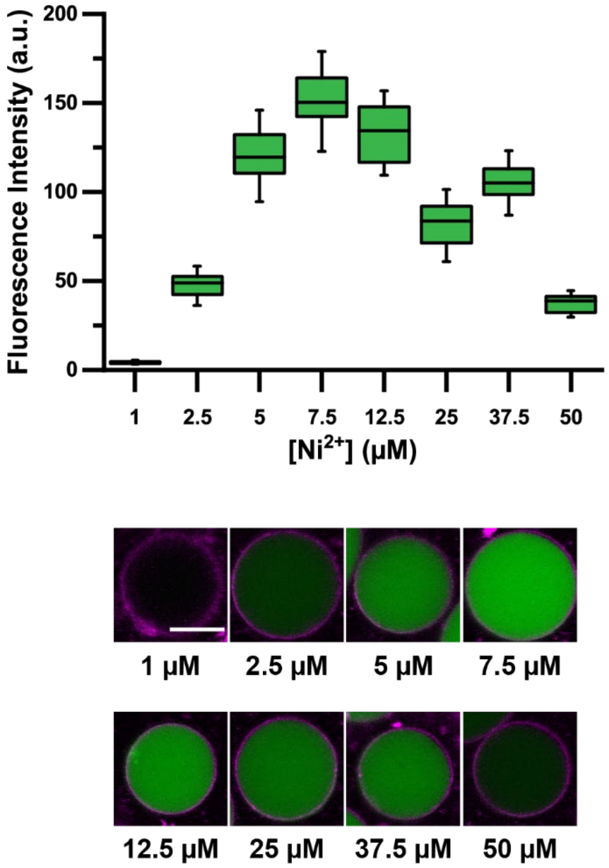

Supplementary Figure 6. The dependency of the amount of sfGFP (100 nM) loaded inside coacervates on the concentration of Ni^{2+} with an amylose mass ratio of 2:0.8:0.2 of Q:Cm:NTA as determined by confocal microscopy. For each condition the same settings were used on the microscope. Top) Boxplot showing the fluorescence intensity for each condition. $N \geq 35$ per concentration. Bottom) Confocal images of each condition analyzed. Scale bar: 10 µm. Polymer membrane stained with Nile Red (Purple). From 1 to 7.5µM of Ni^{2+} there is more NTA compared to Ni^{2+}, so all Ni^{2+} will we taken up into the coacervate and bind to the NTA. From 7.5µM to 50 µM Ni^{2+}, an excess of Ni^{2+} is present compared to NTA. Therefore there will be increasingly amounts of Ni^{2+} in solution which can still interact with the his-tagged proteins. This decreases the driving force for the uptake of the His-tagged protein and thus yields a lower amount localized within the coacervate.

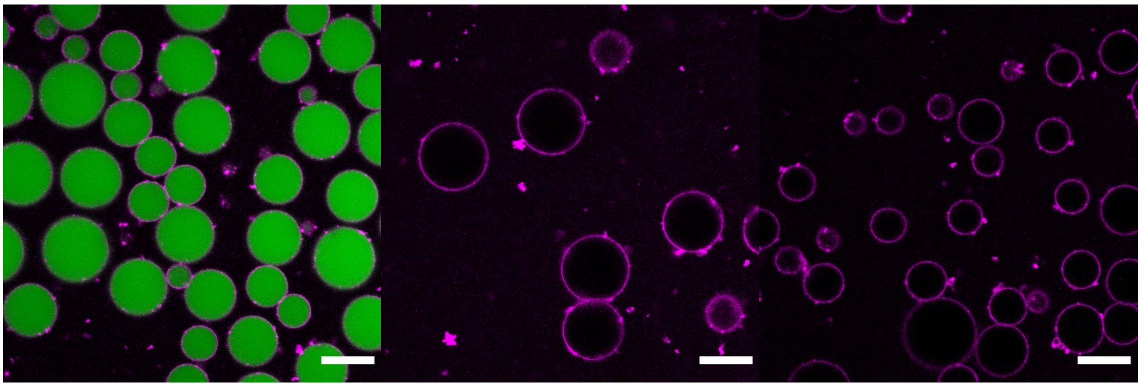

Supplementary Figure 7. Uncropped confocal images of sfGFP-His with Ni, sfGFP with Ni and sfGFP-His, respectively (see main text Figure 2a). Scale bar: 20 µm, polymer membrane stained with Nile red (Purple).

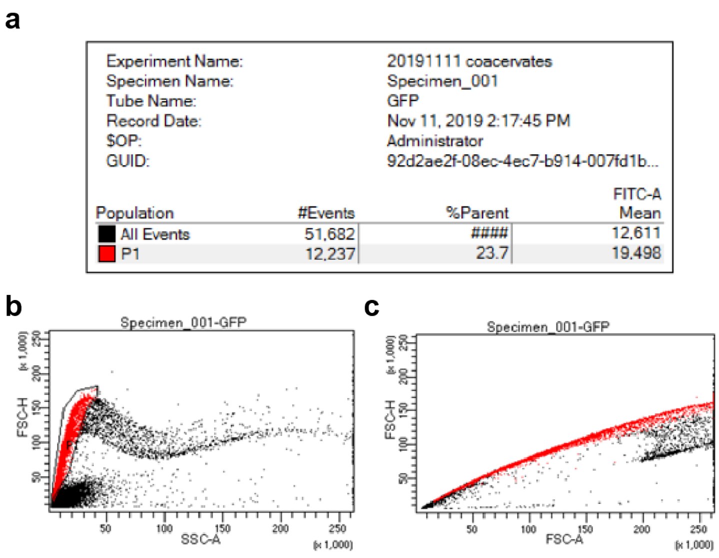

Supplementary Figure 8. Overview of the FACS settings used to select for the coacervates. a) Population statistics of the measurement**.** b, c) Gate settings to select for single particles based on the forward vs the side scatter, selected population shown in red, i.e. droplet containing a single coacervate protocell. Black data points represent droplets containing multiple protocells, terpolymer aggregates or noise. b) Forward scatter height vs side scatter area. c) Forward scatter Height vs forward scatter Area.

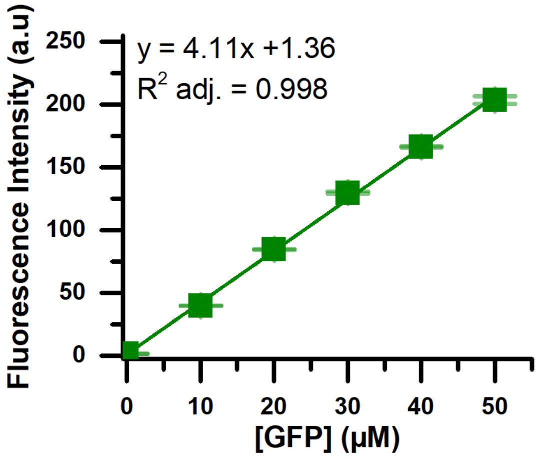

Supplementary Figure 9. The reference curve of sfGFP protein concentration versus fluorescence intensity. For each data point three different images were obtained, error bars represent the standard deviation. The fluorescence intensity was determined with ImageJ.

sfGFP-His

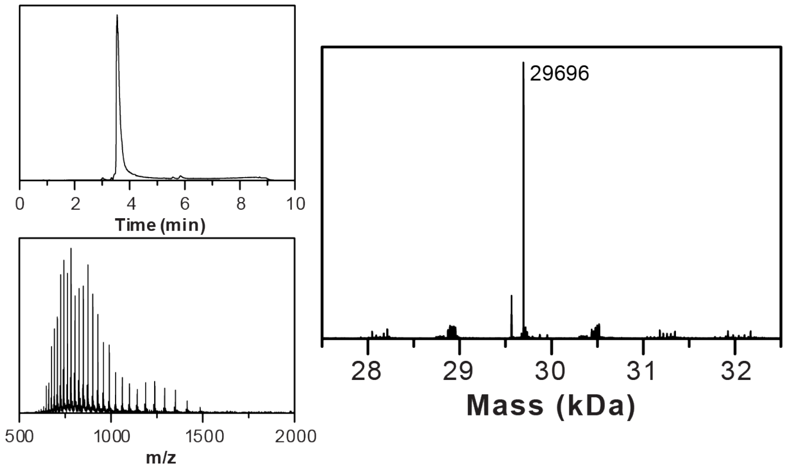

-30GFP-His

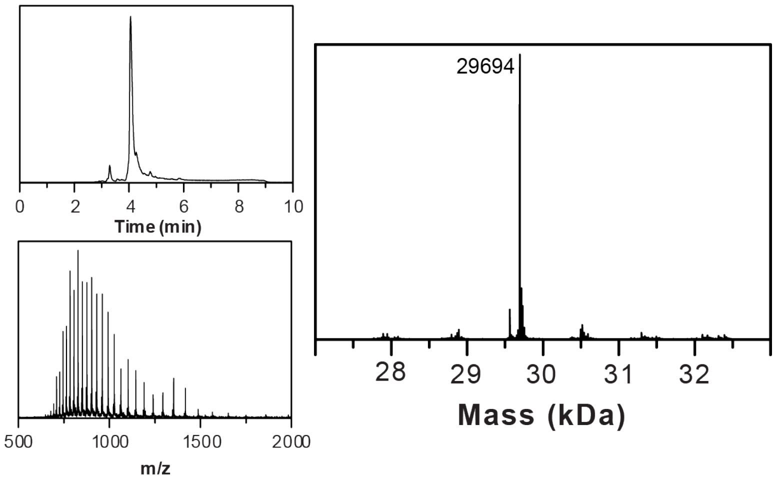

+36GFP-His

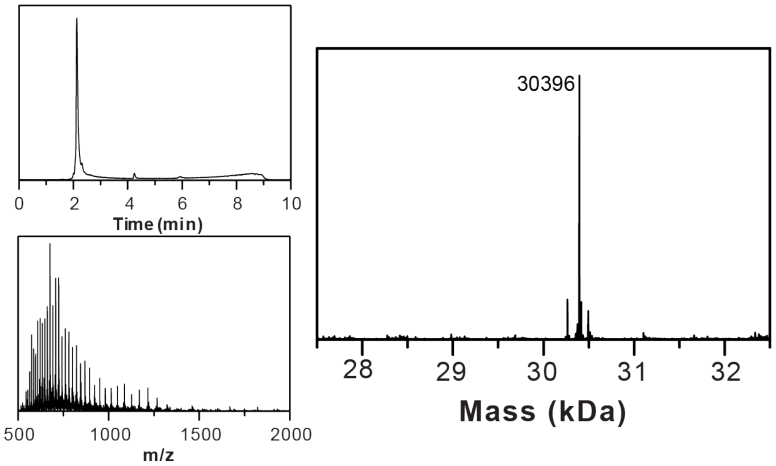

Supplementary Figure 10. LC-MS Q-TOF analysis of each GFP-His variant. For each protein, the total ion count chromatogram (top left), the m/z spectrum (bottom left), and the resulting mass spectrum (right) are shown. The theoretical mass, as calculated using the protein sequence on the online tool ProtParam (ExPASy), are 29716 Da for sfGFP-His, 29714 Da for -30GFP-His , and 30416 Da for +36GFP-His. All the experimental molecular weights are 20 Da lower, which is expected due to chromophore maturation.

sfGFP

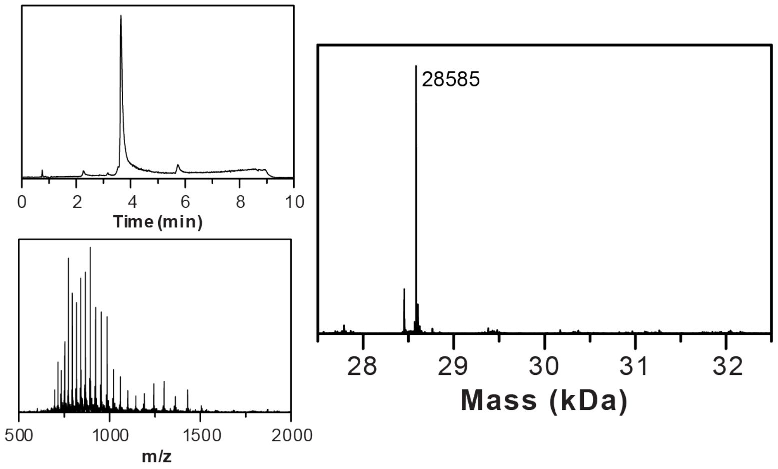

-30GFP

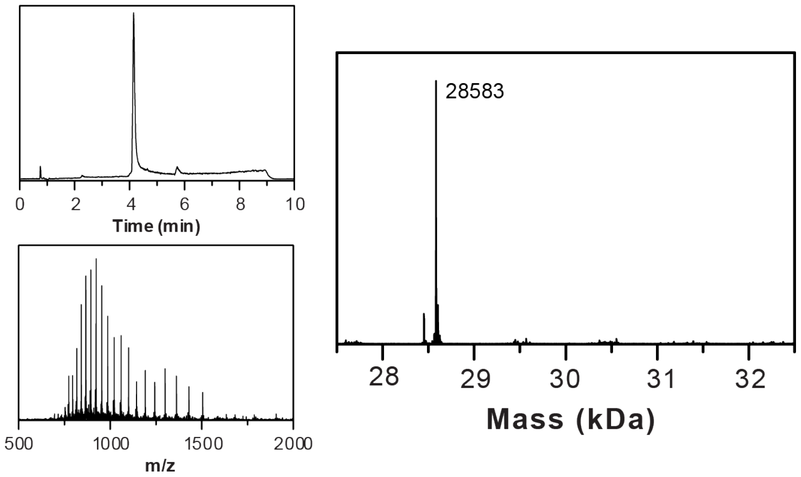

+36GFP

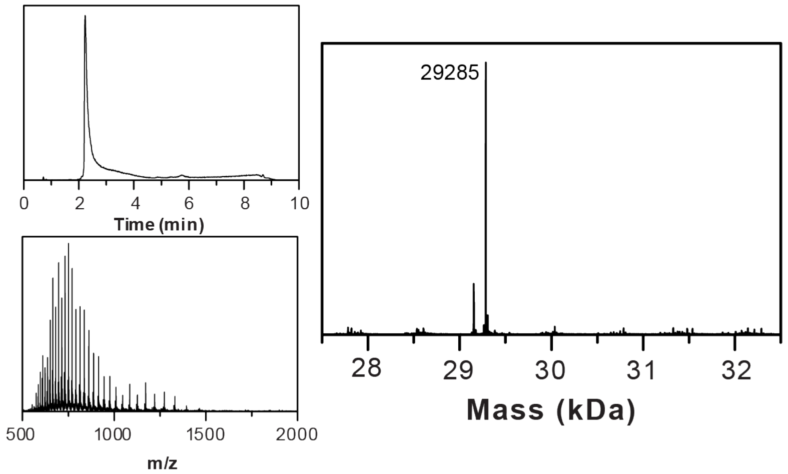

Supplementary Figure 11. LC-MS Q-TOF analysis of each GFP variant after TEV cleavage. For each protein, the total ion count chromatogram (top left), the m/z spectrum (bottom left), and the resulting mass spectrum (right) are shown. The theoretical mass, as calculated using the protein sequence on the online tool ProtParam (ExPASy), are 28585Da for sfGFP, 292853 Da for -30GFP , and 30285 Da for +36GFP, resulting in a loss of 1091 Da for each variant, which corresponds to the histidine tag.

sfGFP:

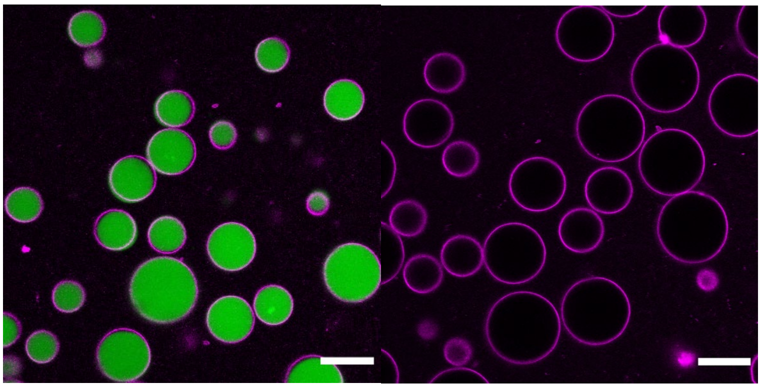

-30GFP:

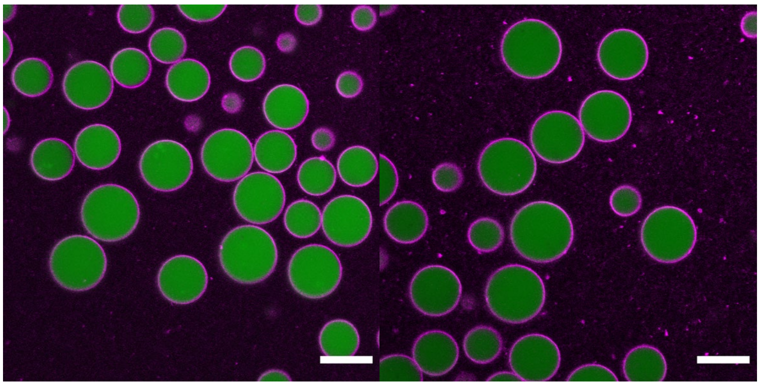

+36GFP:

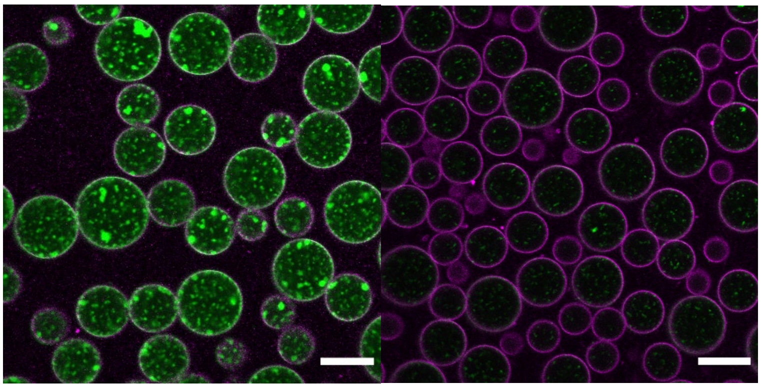

Supplementary Figure 12. The uncropped confocal images of coacervates mixed with GFP variants with histidine tags (left) and without histidine tags (right). Scale bar: 20 µm, polymer membrane stained with Nile Red (Purple). (Main text Figure 3b)

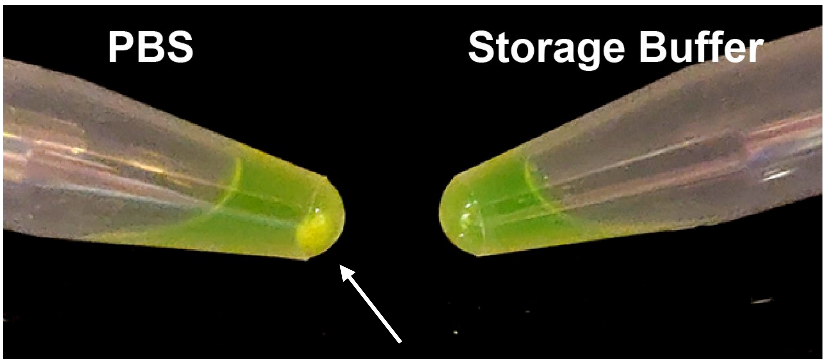

Supplementary Figure 13. Aggregation behavior of +36GFP upon dissolving into a low salt buffer (PBS, 150 mM total ionic strength) compared to a high salt buffer (Storage Buffer, 600 mM total ionic strength).

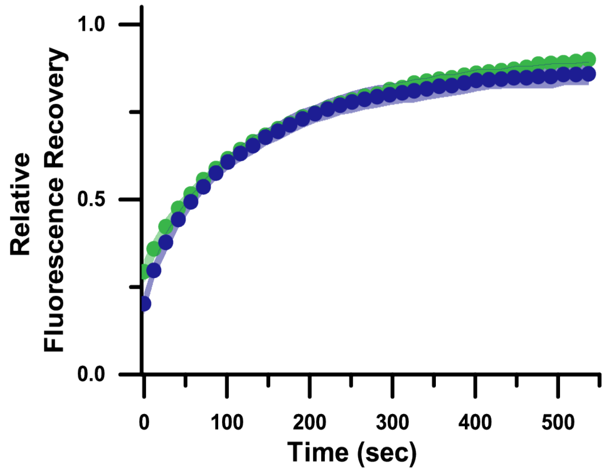

Supplementary Figure 14. The continuous x-axis of the FRAP analysis of sfGFP-His (green) and -30GFP-His (blue), main text Figure 3d.

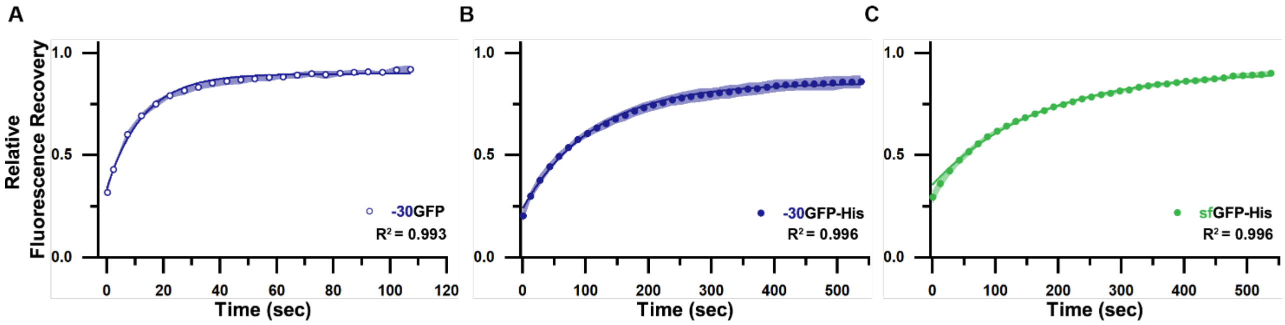

Supplementary Figure 15. The FRAP analysis of -30GFP(A) , -30GFP-His(B) and sfGFP-His (C), with the fitted curves used to calculate the apparent diffusion constant, main text Figure 3d.

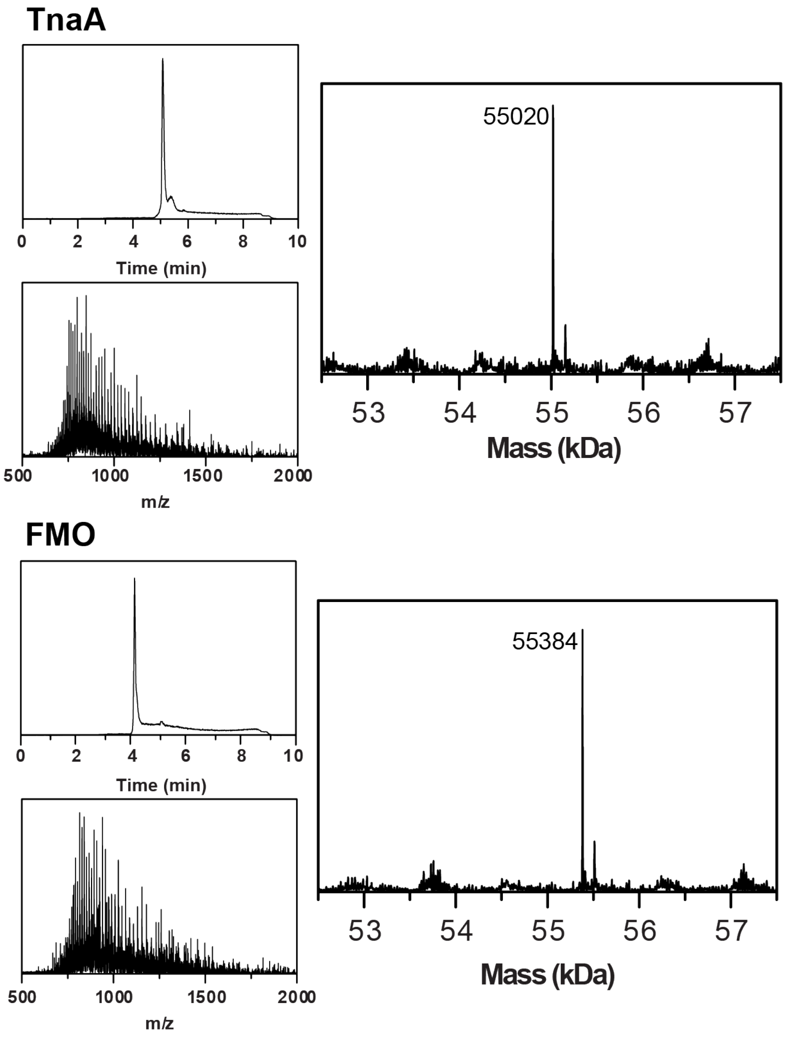

Supplementary Figure 16. LC-MS Q-TOF analysis of each enzyme. For each, the total ion count chromatogram (top left), the m/z spectrum (bottom left), and the resulting mass spectrum (right) are shown. The theoretical mass, as calculated using the protein sequence on the online tool ProtParam (ExPASy), are 55151 Da for TnaA and 55515 Da for FMO. Both are 131 Da lower due to removal of the N terminal methionine residue.

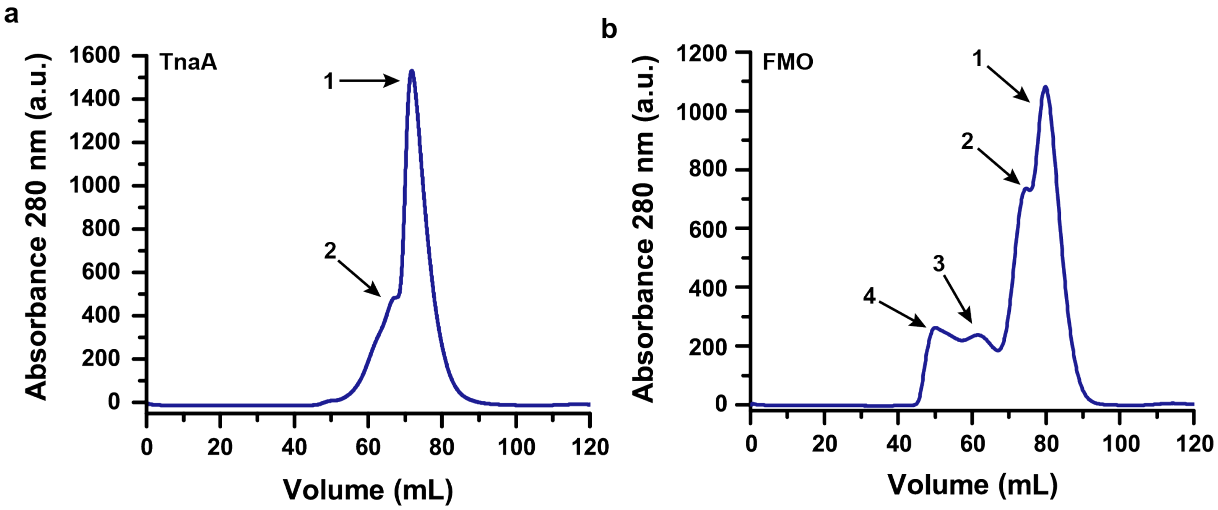

Supplementary Figure 17. Chromatogram of TnaA and FMO enzymes purified with a HiLoad Superdex 26/600 200 pg, 1mL/min, 4°C. A) Chromatogram of TnaA, 1. Monomer, 2. Dimer. B) Chromatogram of FMO, 1. Monomer, 2. Dimer, 3 and 4. Enzyme lacking the cofactor FAD.

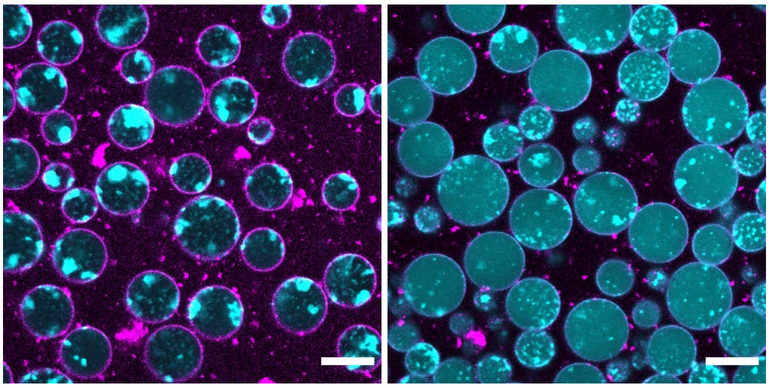

Supplementary Figure 18. Uncropped confocal images of TnaA-Cy5 + unlabeled FMO (Left) and FMO-Cy5 + unlabeled TnaA (Right). Scale bar: 20 µm. On average, half of the proteins were labeled with a dye. Membrane stained with Nile Red (Purple) (Main text Figure 4c)

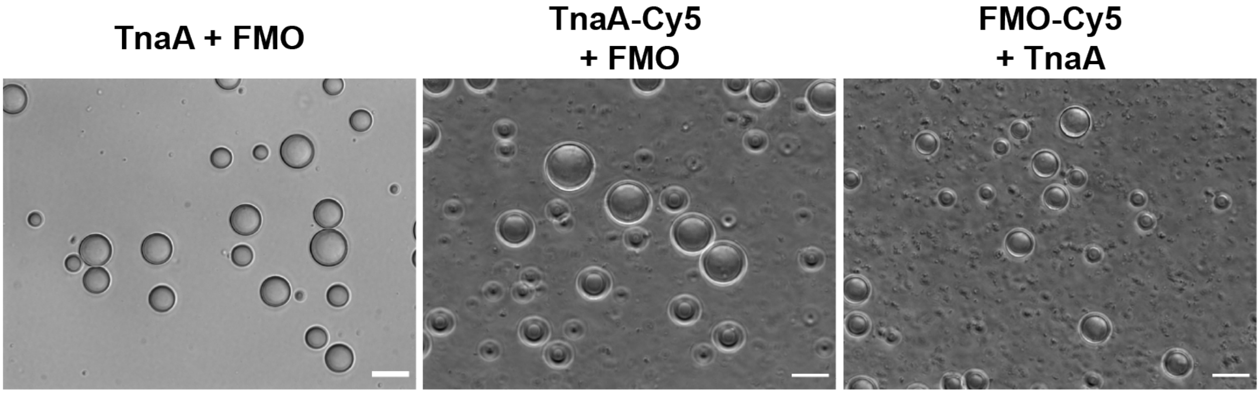

Supplementary Figure 19. Bright-field microscopy images of unlabeled TnaA + FMO (left), TnaA=Cy5 + unlabeled FMO (middle) and FMO-Cy5 + unlabeled TnaA(right). Scale bar: 20 µm.

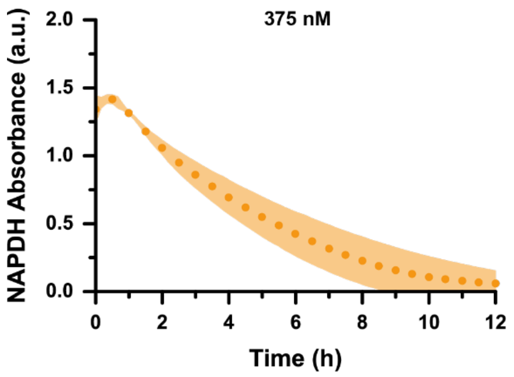

Supplementary Figure 20. NADPH absorbance at 340 nm over time for Cy5 labeled TnaA and FMO. Conditions are the same as in Figure 4f. Three individual batches were made for each condition, error represented by the shaded area. For clarity of the graph, only 1/3rd of all collected data points are shown.

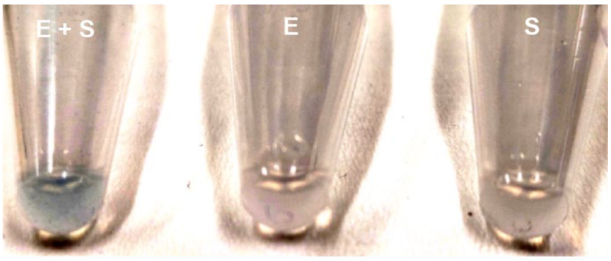

Supplementary Figure 21. Indigo production inside coacervates after an overnight reaction. Left to right: Coacervates containing 1:2 ratio of TnaA:FMO (750 nM total) + 0.5 mM substrate (E + S), Enzymes only (E), 0.5 mM substrate (S)

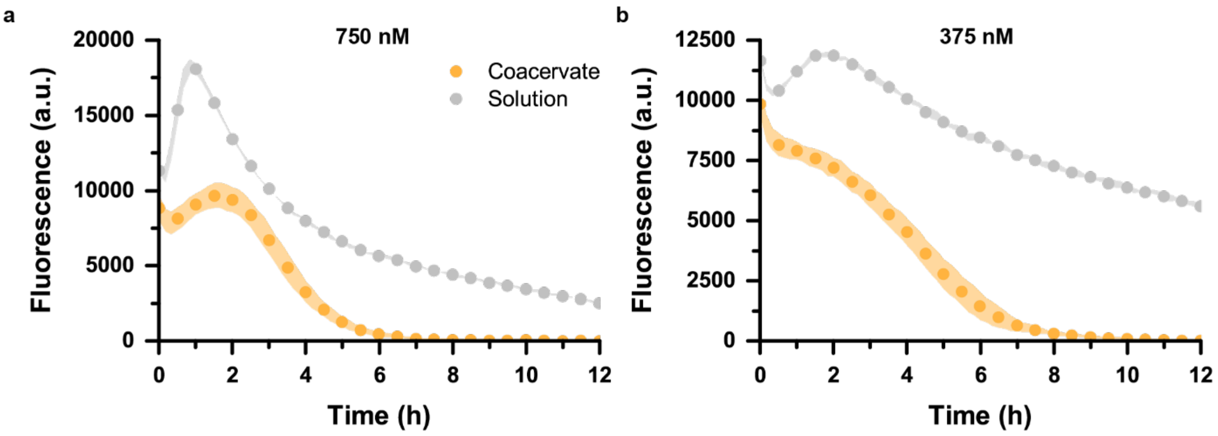

Supplementary Figure 22. Indoxyl fluorescence measured over time, excitation: 375 nm, emission: 470 nm, 20 nm bandwidth. Coacervates are represented in Orange and solution in gray. n = 3, individual batches of coacervates for each condition, error represented by the shaded area. The level of 10000 fluorescence at the start is due to the presence and absorbance of NADPH. For clarity of the graph, only 1/3rd of all collected data points are shown. a) 250 nM TnaA + 500 nM FMO b) 125 nM TnaA + 250 nM FMO (Main text Figure 4e, f)

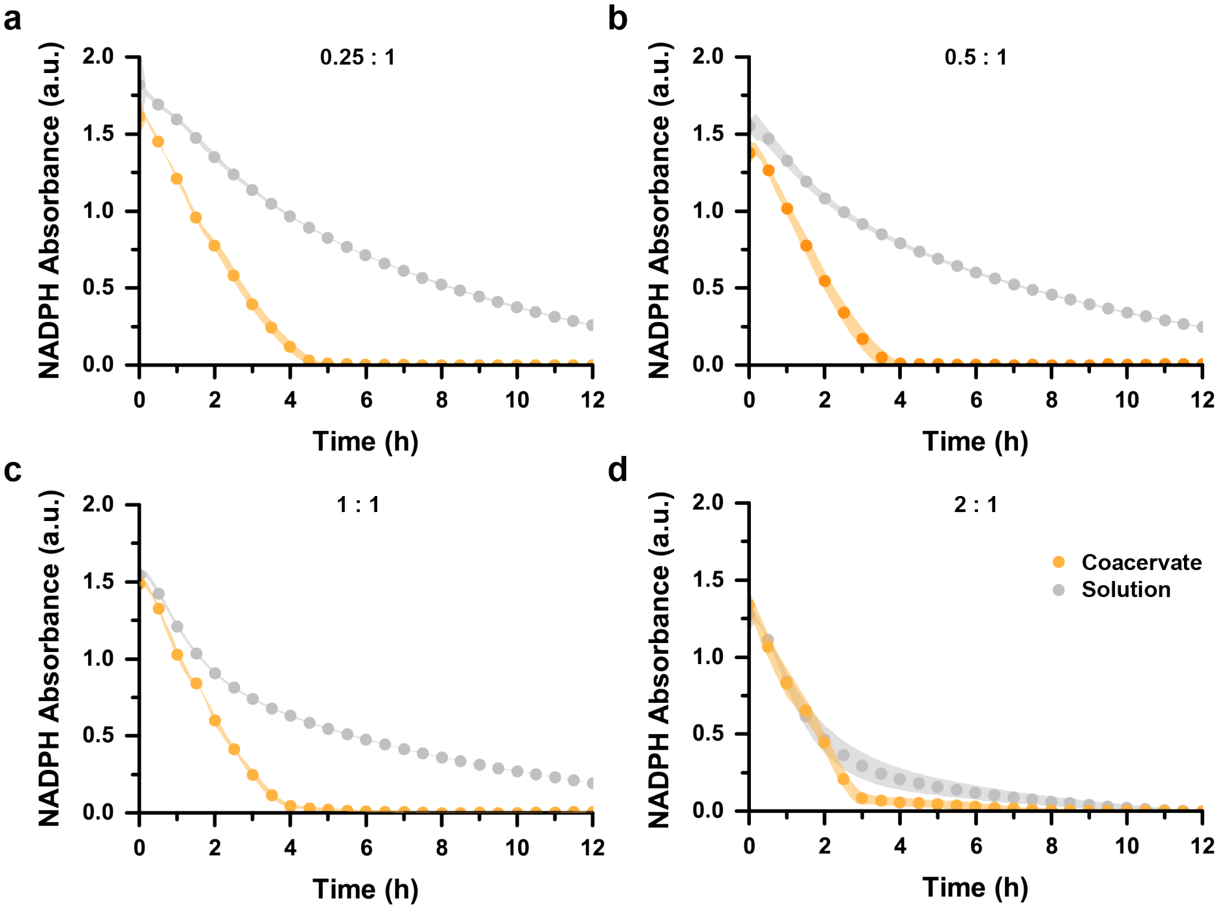

Supplementary Figure 23. NADPH absorbance at 340 nm over time for different ratios of TnaA:FMO, with FMO constant at 500 nM, either loaded into coacervates (orange) or in solution(gray). Three individual batches were made for each condition, error represented by the shaded area. For clarity of the graph, only 1/3rd of all collected data points are shown. a) 125:500 nM b) 250:500 nM c) 500:500 nM d) 1000:500 nM of TnaA:FMO respectively.

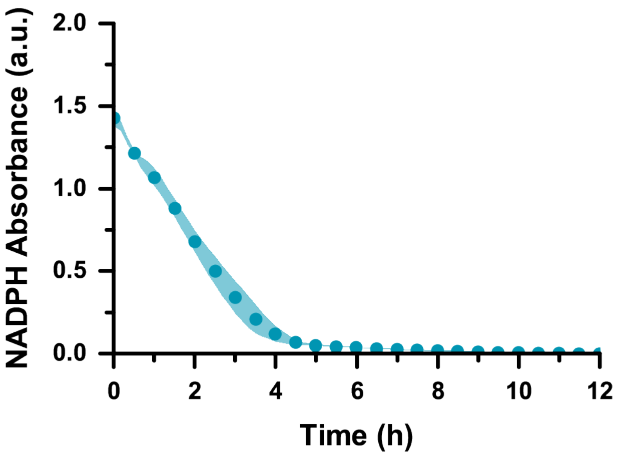

Supplementary Figure 24. NADPH consumption measured by the absorbance at 340 nm for 750 nM enzyme, 0.5:1 ratio of TnaA:FMO, in absence of Ni^{2+}. Three individual batches were made for each condition, error represented by the shaded area. For clarity of the graph, only 1/3rd of all collected data points are shown.

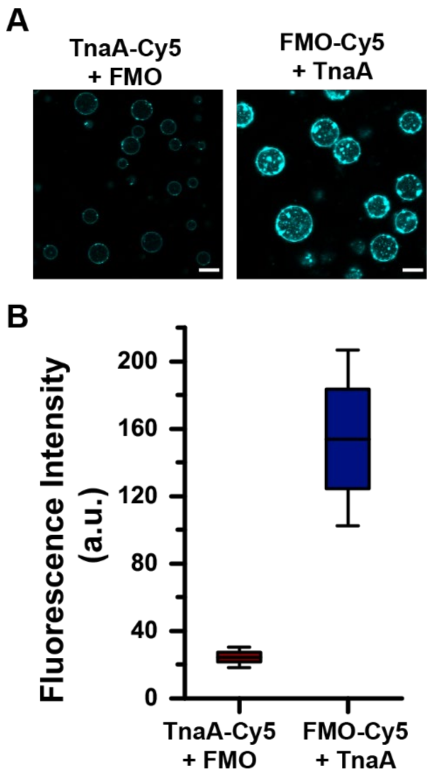

Supplementary Figure 25. The uptake of TnaA-Cy5 or FMO-Cy5 into coacervates without Ni^{2+} present. A) Confocal micrographs of representative enzyme loaded coacervates. TnaA-Cy5 + unlabeled FMO (left), FMO-Cy5 + unlabeled TnaA (right). Scale bar: 20 µm. B) Box plot analysis of the loading of fluorescently labelled enzymes inside the coacervates. For all cases n > 19.

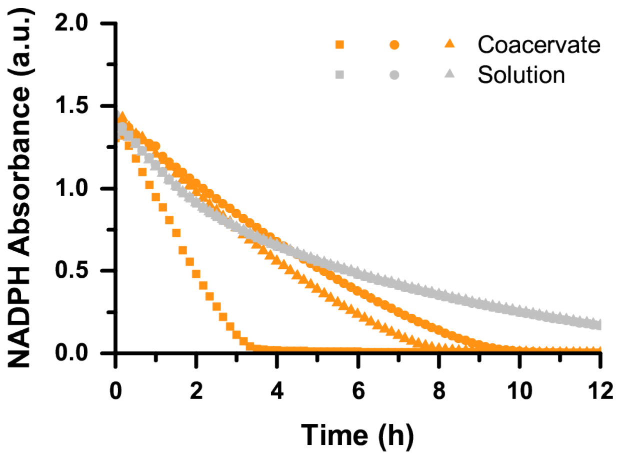

Supplementary Figure 26. The effect of inactive TnaA on the enzymatic cascade due to solution temperatures below RT for the enzyme. NADPH consumption measured by the absorbance at 340 nm for 750 nM enzyme, 0.5:1 ratio of TnaA:FMO. Coacervates in orange, solution in gray. Three individual batches were made for each condition.

sfGFP-His:

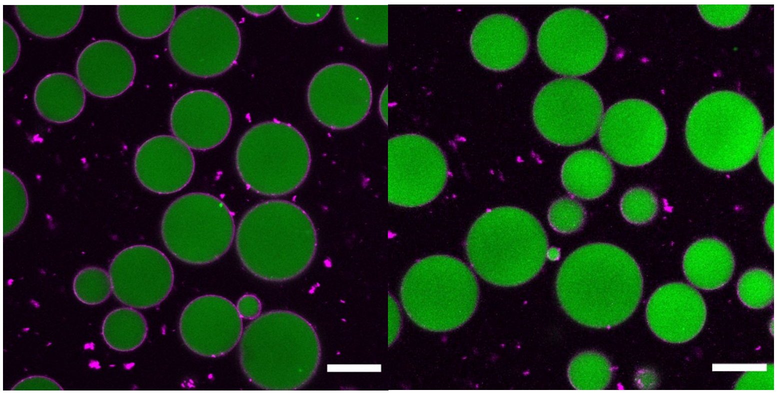

-30GFP-His:

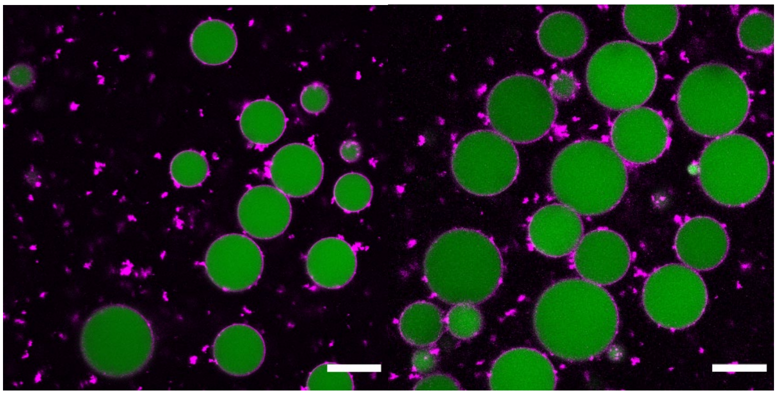

Supplementary Figure 27. Uncropped confocal images of 100 nM sfGFP-His and -30GFP-His with (left) and without TEV protease (right). Membrane stained with Nile Red (Purple), scale bar: 20 µm. Main text figure 5b.

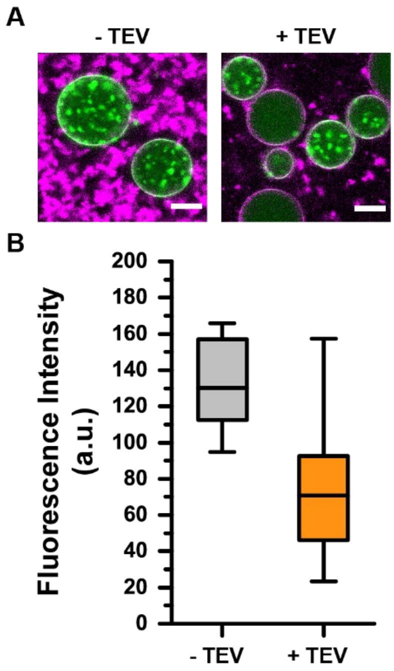

Supplementary Figure 28. Fluorescence intensity inside the coacervate droplet incubated with (orange) and without (gray) TEV protease for 100 nM of +36GFP. Membrane stained with Nile red (purple), scale bar: 10 µm.

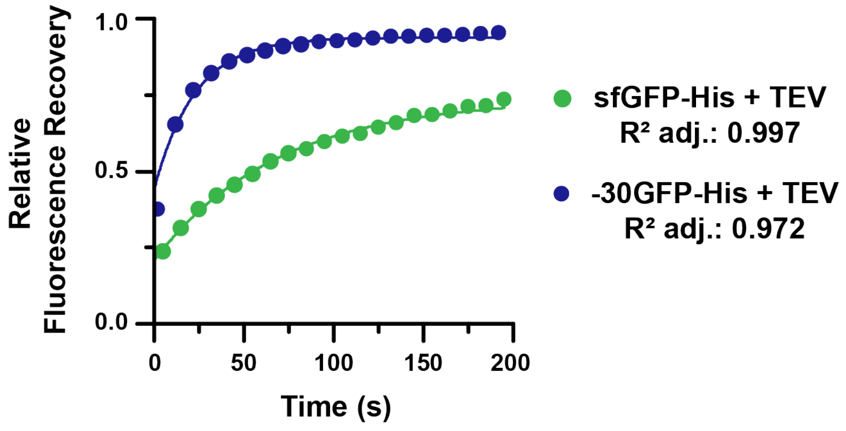

Supplementary Figure 29. The FRAP analysis of sfGFP-His (green), -30GFP-His(blue) with the fitted curves used to calculate the apparent diffusion constant, main text Figure 5c.

With TEV

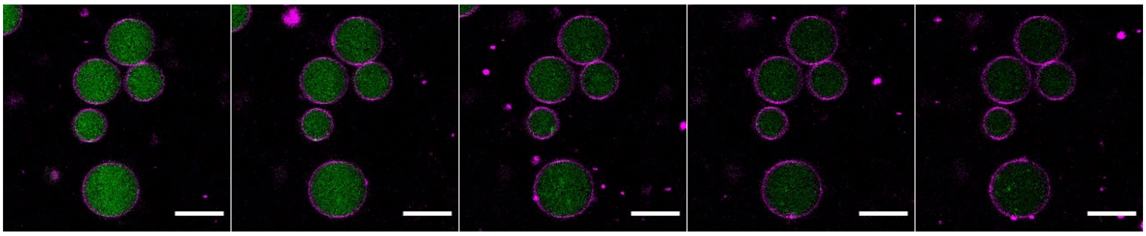

Without TEV

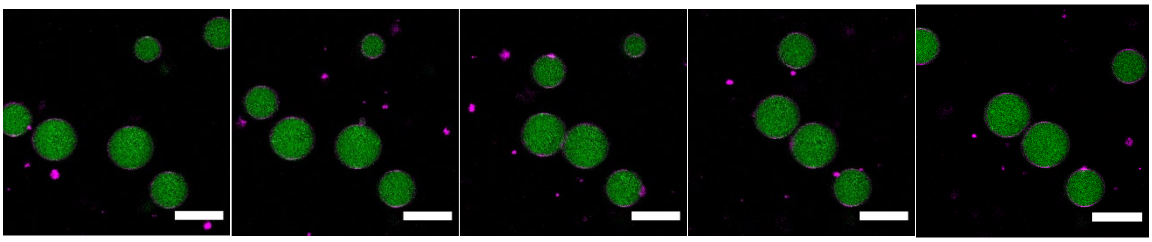

Supplementary Figure 30. The uncropped confocal images of the time trace of 250 nM of sfGFP-His with (top) and without TEV protease (bottom). Scale bar: 20 µm, membrane stained with Nile Red (purple)

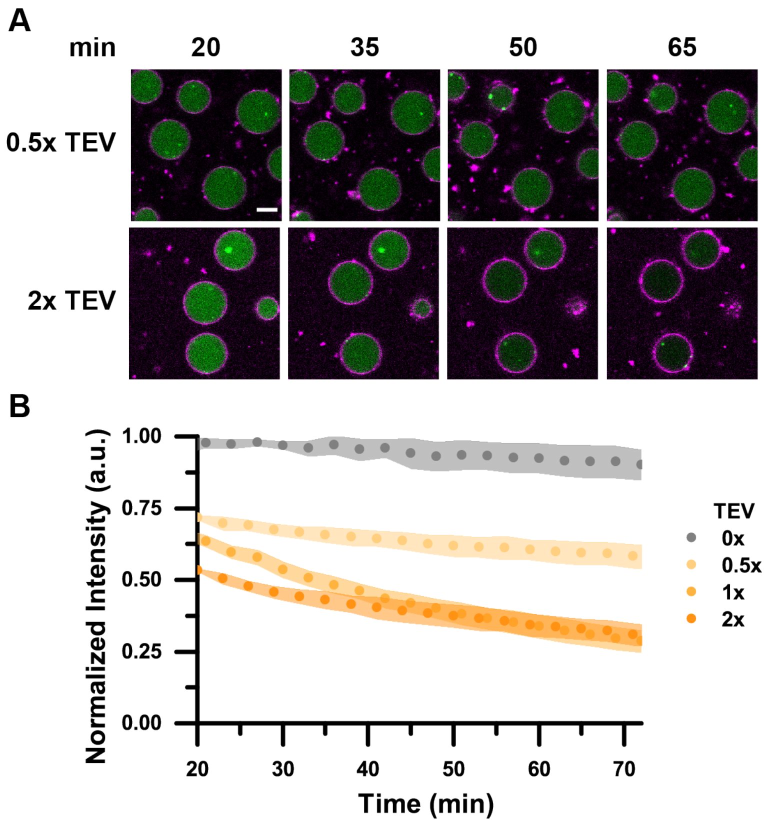

Supplementary Figure 31. Release profile of 250 nM sfGFP determined by confocal microscopy. A) Time series of representative protocells. Membrane stained with Nile Red (Purple), scale bar: 10 µm. B) Normalized fluorescence intensity of n >20 protocells extracted from confocal time series. 1x and 0x TEV is the same data shown in main text figure 5d. The similar release rate for 1x and 2x TEV around 50 minutes can be explained by substrate depletion, e.g. amount of sfGFP present inside the protocell, and the diffusivity of the His-tagged protein in the NiNTA functionalized coacervates (main text Figure 3).

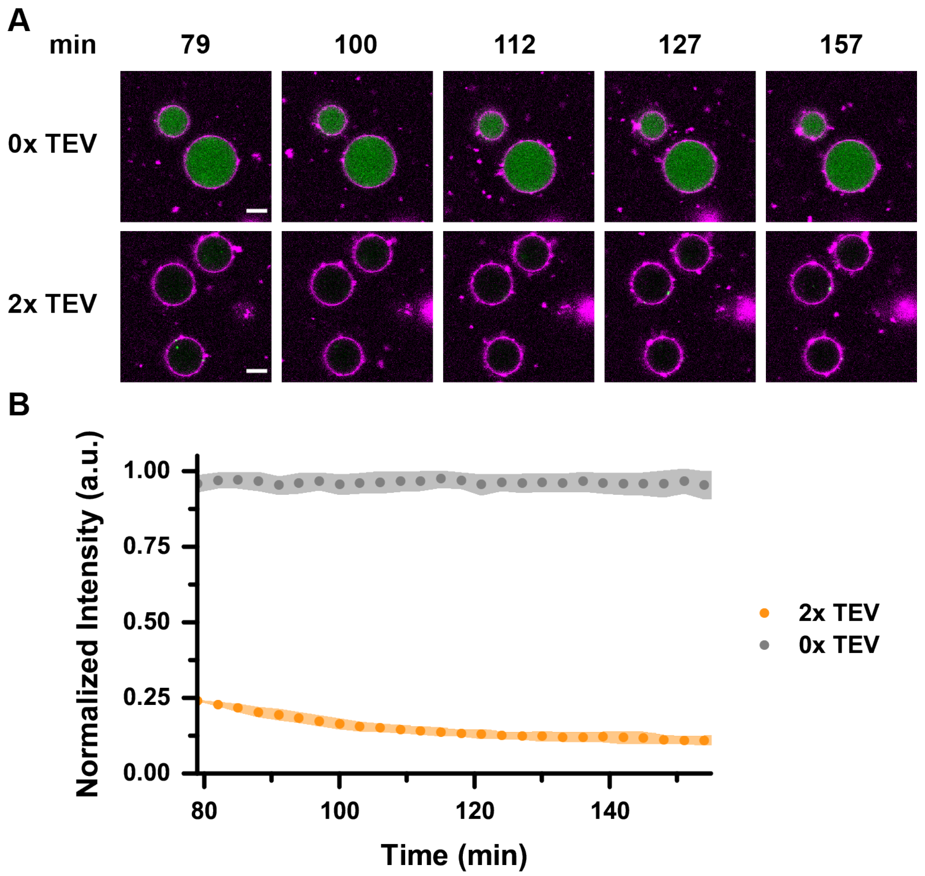

Supplementary Figure 32. Release profile of 250 nM sfGFP determined by confocal microscopy. A) Time series of representative protocells. Membrane stained with Nile Red (Purple), scale bar: 10 µm. B) Normalized fluorescence intensity of n >20 protocells extracted from confocal time series.

Supplementary References:

1. Mason, A. F., Buddingh', B. C., Williams, D. S. & van Hest, J. C. M. Hierarchical Self-Assembly of a Copolymer-Stabilized Coacervate Protocell. *J. Am. Chem. Soc.* **139**, 17309–17312 (2017).
2. Couffin, A., Delcroix, D., Martín-Vaca, B., Bourissou, D. & Navarro, C. Mild and Efficient Preparation of Block and Gradient Copolymers by Methanesulfonic Acid Catalyzed Ring-Opening Polymerization of Caprolactone and Trimethylene Carbonate. *Macromolecules* **46**, 4354–4360 (2013).
3. Zou, J. *et al.* A Facile Glovebox-Free Strategy To Significantly Accelerate the Syntheses of Well-Defined Polypeptides by N-Carboxyanhydride (NCA) Ring-Opening Polymerizations. *Macromolecules* **46**, 4223–4226 (2013).
