# Supplementary material for: Programmed spatial organization of biomacromolecules into discrete, coacervate-based protocells
Source: Nat Commun. 2020 Dec 8;11:6282. doi: 10.1038/s41467-020-20124-0 (PMC7722712; doi:10.1038/s41467-020-20124-0)
Supplement: Supplementary file 3 — Reporting Summary [file 41467_2020_20124_MOESM3_ESM.pdf]

nature research

Corresponding author(s): Alexander F. Mason, Jan C.M. van Hest

Last updated by author(s): 21/10/20

Reporting Summary

Nature Research wishes to improve the reproducibility of the work that we publish. This form provides structure for consistency and transparency in reporting. For further information on Nature Research policies, see Authors & Referees and the Editorial Policy Checklist .

Statistics

For all statistical analyses, confirm that the following items are present in the figure legend, table legend, main text, or Methods section.

n/a	Confirmed	
☐	☒	The exact sample size (*n*) for each experimental group/condition, given as a discrete number and unit of measurement
☐	☒	A statement on whether measurements were taken from distinct samples or whether the same sample was measured repeatedly
☒	☐	The statistical test(s) used AND whether they are one- or two-sided<br>*Only common tests should be described solely by name; describe more complex techniques in the Methods section.*
☒	☐	A description of all covariates tested
☒	☐	A description of any assumptions or corrections, such as tests of normality and adjustment for multiple comparisons
☐	☒	A full description of the statistical parameters including central tendency (e.g. means) or other basic estimates (e.g. regression coefficient) AND variation (e.g. standard deviation) or associated estimates of uncertainty (e.g. confidence intervals)
☒	☐	For null hypothesis testing, the test statistic (e.g. *F*, *t*, *r*) with confidence intervals, effect sizes, degrees of freedom and *P* value noted<br>*Give P values as exact values whenever suitable.*
☒	☐	For Bayesian analysis, information on the choice of priors and Markov chain Monte Carlo settings
☒	☐	For hierarchical and complex designs, identification of the appropriate level for tests and full reporting of outcomes
☒	☐	Estimates of effect sizes (e.g. Cohen's *d*, Pearson's *r*), indicating how they were calculated

Our web collection on statistics for biologists contains articles on many of the points above.

Software and code

Policy information about availability of computer code

Data collection	Tecan Spark Platereader: Tecan SparkControl v2.1<br>Zeiss Laser Scanning Microscope: Zen 2009<br>Leica DMI8-CS Microscope: LAS X<br>NMR: Bruker TopSpin 4.0.6<br>BD Aria III FACS: FACSDiva<br>GE Healthcare ImageQuant Capute v1.0.2<br>NanoDrop ND-1000 v3.5.2
Data analysis	All numerical data was analyzed and visualized with Origin 2019, 9.6.0.172, Academic (OriginLab).<br>For all microscopy data, FIJI (ImageJ) version 1.52p was used. (https://imagej.net/Fiji)<br>In the case of NMR the data processing was performed with TopSpin 4.0.6

For manuscripts utilizing custom algorithms or software that are central to the research but not yet described in published literature, software must be made available to editors/reviewers. We strongly encourage code deposition in a community repository (e.g. GitHub). See the Nature Research guidelines for submitting code & software for further information.

Data

Policy information about availability of data

All manuscripts must include a data availability statement. This statement should provide the following information, where applicable:

- Accession codes, unique identifiers, or web links for publicly available datasets
- A list of figures that have associated raw data
- A description of any restrictions on data availability

Full experimental details and data are provided in the Supplementary Information. All source data is available upon reasonable request from the corresponding author.

Field-specific reporting

Please select the one below that is the best fit for your research. If you are not sure, read the appropriate sections before making your selection.

- [x] Life sciences
- [ ] Behavioural & social sciences
- [ ] Ecological, evolutionary & environmental sciences

For a reference copy of the document with all sections, see nature.com/documents/nr-reporting-summary-flat.pdf

Life sciences study design

All studies must disclose on these points even when the disclosure is negative.

Sample size	No statistical method was used to determine the sample size. For all fluorescence intensity measurement at least 20 particles in at least three different fields of view were analyzed. For the FRAP measurement, three different protocells in different fields of view were analyzed. For all enzymatic studies, triplicates of freshly made sample were made for each sample. In the case of TEV release, five different fields of view were analyzed per sample.
Data exclusions	For all microscopy experiments, particles that are not fully in the focal plane were excluded. Regarding time series, particles that move out of the field of view or focal plane were not used for analysis. In the case of FACS experiments, any data point which had the maximum value of the detector was removed from the data set.
Replication	All experiments were reproducible with similar results.
Randomization	None of the experiments involve animal or human participants , therefore not applicable.
Blinding	None of the experiments involve animal or human participants , therefore not applicable.

Reporting for specific materials, systems and methods

We require information from authors about some types of materials, experimental systems and methods used in many studies. Here, indicate whether each material, system or method listed is relevant to your study. If you are not sure if a list item applies to your research, read the appropriate section before selecting a response.

Materials & experimental systems

n/a	Involved in the study	
x		Antibodies
x		Eukaryotic cell lines
x		Palaeontology
x		Animals and other organisms
x		Human research participants
x		Clinical data

Methods

n/a	Involved in the study	
x		ChIP-seq
	x	Flow cytometry
x		MRI-based neuroimaging

Flow Cytometry

Plots

Confirm that:

☒ The axis labels state the marker and fluorochrome used (e.g. CD4-FITC).

☒ The axis scales are clearly visible. Include numbers along axes only for bottom left plot of group (a 'group' is an analysis of identical markers).

☒ All plots are contour plots with outliers or pseudocolor plots.

☒ A numerical value for number of cells or percentage (with statistics) is provided.

Methodology

Sample preparation	Coacervate protocells were made in the same manner as all other experiments. Each sample was diluted two times before loaded into the machine to lower the chances of multiple particles per event.
Instrument	Aria III (BD Biosciences)
Software	FACSDiva
Cell population abundance	Sorting was applied, therefore not applicable.
Gating strategy	Single coacervates were selected based the forward scatter and side scatter

☒ Tick this box to confirm that a figure exemplifying the gating strategy is provided in the Supplementary Information.
